# An analysis of the rise of English-taught programmes in the North Macedonian context: the impact of policy spillover

**DOI:** 10.1007/s10993-026-09801-w

**Published:** 2026-08-12

**Authors:** Dogan Yuksel, Peter Wingrove, Marion Nao, Beatrice Zuaro, Anna Kristina Hultgren

**Affiliations:** 1 Faculty of Wellbeing, Education & Language Studies, The Open University, Milton Keynes, UK; 2 Kocaeli University, Kocaeli, Turkey; 3 Chalmers University of Technology, Gothenburg, Sweden; 4 University of Copenhagen, Copenhagen, Denmark

**Keywords:** Language policy and planning, North Macedonia, English medium instruction, Steering at a distance, Higher education governance, Social process tracing

## Abstract

This study examines the proliferation of English-medium instruction (EMI) within North Macedonia’s higher education system, a context emblematic of broader regional trends towards Europeanisation in the Western Balkans. Moving beyond the steering at a distance framework (Kickert, 1995), we analyse EMI development through the lens of post-socialist state-building and marketisation. Employing social process tracing (Kaas et al., 2025), we analysed higher education policy documents and regulations, complemented by interviews with key decision-makers to map the trajectory of English-taught programmes. Contrary to the assumption that EMI growth is driven by explicit language planning, our findings reveal a consistent pattern of policy spillover. We demonstrate that while no national policy explicitly mandated EMI, successive reforms—aimed variously at multilingual inclusion, privatisation, marketisation, Bologna harmonisation, and institutional liberalisation—inadvertently created the conditions for EMI to thrive. This study contributes to the literature by reframing EMI institutionalisation not merely as a pedagogical or linguistic goal, but as an institutionalised by-product of broader governance transformations. These findings offer significant implications for policymakers and researchers, suggesting that effective language planning requires analysing the unintended consequences of macro-level governance reforms.

## Introduction

English medium instruction (EMI) has become a defining feature of higher education systems across Europe, driven by internationalisation agendas, Europeanisation pressures, and neo-liberal market forces (Galloway et al., 2020; Wingrove et al., 2025, 2026). While the expansion of EMI in Western and Northern Europe is well-documented (e.g., Hultgren, 2024), the trajectory of English-taught programmes in the Western Balkans offers a distinct and under-researched perspective (Wingrove, 2025). In North Macedonia—a post-conflict society navigating complex ethnic, linguistic, and governance dynamics—EMI has proliferated across both public and private universities. However, unlike contexts where EMI results from explicit top-down language planning, its growth in North Macedonia appears to be an institutional response to broader structural changes. This raises a critical question regarding the originality of EMI policy analysis: how do governance reforms designed for political and economic state-building inadvertently function as de facto language policies?

This study addresses this question by examining the drivers behind the rise of EMI in North Macedonian higher education. We propose that EMI in this context is best understood through the lens of policy spillover—a process where policies aimed at one objective have significant, unintended consequences in another domain (Barzelay & Gallego, 2006). Specifically, we examine how the massification and privatisation of the sector (Sokoli et al., 2025), post-Ohrid Framework Agreement commitments to linguistic and ethnic inclusion (Pollozhani, 2022), and the country’s aspiration for European Union integration (Besimi, 2025) collectively created a fertile ground for the rise of EMI, even in the absence of a national strategy explicitly mandating it.

Theoretically, we argue that these reforms were driven by the logic of steering at a distance (Kickert, 1995)—a governance model where the state retreats from direct control, granting institutions greater operational autonomy while retaining influence through accountability mechanisms and performance indicators (Lingard, 1996; Marginson, 1997). Under this model, the government becomes just one of many actors shaping institutional decisions, often exerting control from the periphery rather than the centre. In the North Macedonian context, this steering—manifested through liberalisation (Sharlamanov & Petreska, 2023) and privatisation (Sokoli et al., 2025)—fostered the specific conditions conducive to the adoption and institutionalisation of EMI.

Methodologically, this study employs social process tracing (SPT) (Kaas et al., 2025; Zuaro et al., 2025) to uncover the causal mechanisms enabling this development. Unlike traditional positivist process tracing, SPT adopts an interpretivist focus, capturing not only the moves performed by actors but also the meanings they ascribe to them and how the underlying social context shapes these causal processes (Kaas et al., 2025). This approach is particularly suited to our inquiry, as it recognises that policy outcomes are mediated by human interpretation. It allows us to trace how university leaders and policymakers made sense of macro-level governance reforms and translated them into specific institutional actions (Zuaro et al., 2025). Utilising an iterative, abductive design that moves back and forth between process theory and empirics (Schwartz-Shea & Yanow, 2013), we identified key episodes (Steel, 2007) in which governance signals were interpreted as mandates for Englishisation. Through an in-depth analysis of legal documents, university strategies, and semi-structured interviews, we map the social processes through which non-linguistic reforms were re-interpreted as drivers for EMI.

The significance of this research lies in its reframing of the EMI debate. By identifying policy spillover via an SPT approach, we challenge a pervasive assumption in both classical language planning frameworks and mainstream EMI policy research: namely, that language shifts in higher education are the result of deliberate linguistic intent. Classical language planning models conceptualise policy as goal-oriented state intervention (Kaplan & Baldauf, 1997), while much mainstream EMI scholarship frames programme adoption as the outcome of explicit institutional or governmental decisions (Dearden, 2014; Macaro et al., 2018; Rose et al., 2021). Even critical language policy approaches, which interrogate the power relations embedded in such decisions, tend to presuppose that language shifts are driven by identifiable ideological agents acting with deliberate purpose (Phillipson, 1992; Tollefson, 1991).This offers a novel contribution to both higher education and language policy literature by demonstrating how political agreements and quality frameworks—often designed for peace-building or marketisation—can inadvertently facilitate Englishisation. For policymakers, these findings suggest that effective language planning requires a holistic analysis of governance reforms, as the most potent language policies may be hidden within regulations that make no mention of language.

## Review of literature

### The language issue in North Macedonia

North Macedonia is characterised by a high degree of linguistic and ethnic diversity, in which language operates not only as a medium of communication but also as a powerful symbol of political identity and ethnic recognition (Andeva et al., 2022). Macedonian is the official state language, while Albanian—spoken by over 20% of the population—has acquired co-official status in various domains following the Ohrid Framework Agreement (OFA) in 2001. In addition, minority languages such as Turkish, Romani, Serbian, and Bosnian contribute to a complex multilingual landscape, where linguistic rights are intricately tied to local demographics and political negotiations (Trix, 2013). Tensions surrounding language use in education have historically mirrored broader ethnic conflicts, with the issue of Albanian-language higher education representing a particularly contentious point (Czaplinski, 2008).

The constitutional and legal frameworks that regulate language use in education and governance have historically reflected and reproduced inter-ethnic tensions. The 1991 Constitution significantly downgraded the status of ethnic Albanians, resulting in their underrepresentation in public administration and higher education (Ilievski, 2007). The 1995 state-led closure of the unrecognised Albanian-language University of Tetovo was widely interpreted as a form of systemic exclusion, intensifying ethnic mobilisation (Rizova, 2011). These tensions culminated in the 2001 armed conflict, which was subsequently resolved through the OFA—a landmark agreement that introduced important guarantees for linguistic and educational rights.

The OFA mandated that public institutions provide education in minority languages spoken by at least 20% of the population. This principle led to the formal recognition of the State University of Tetovo in 2004, where Albanian became the primary language of instruction (Koksal, 2014). It also laid the groundwork for the establishment of the South East European University (SEEU) in 2001, which offered multilingual education in Albanian, English, and Macedonian, reflecting both conflict resolution and internationalisation goals (OECD Report, 2004).

Building on this legal foundation, Bliznakovski (2013) conceptualises the Macedonian language rights framework as a multi-tiered structure. At one level, languages spoken by at least 20% of the population, such as Albanian and Turkish, may be used in public administration and local governance. At another, more expansive level, the legal framework also recognises the right to access education in minority languages even where the 20% threshold is not met, particularly in early childhood and secondary schooling. These provisions underscore a broader human rights approach to linguistic inclusion and access to education.

Taken together, these developments demonstrate that language-in-education policy in North Macedonia cannot be disentangled from broader questions of ethnic politics, state legitimacy, and decentralised governance. Language decisions often function as proxies for debates over autonomy and power-sharing, making higher education a particularly charged site in the negotiation of national identity and minority rights.

### The higher education system in North Macedonia

The higher education system in North Macedonia has experienced significant structural shifts over the past three decades, shaped by post-socialist liberalisation, ethnic conflict and reconciliation processes, and efforts toward European integration (Spasovski & Pecakovska, 2020). Since the 1990s, this landscape has evolved from a highly centralised and monolingual system into one defined by institutional expansion, linguistic diversification, and the emergence of private and internationalised universities. These transformations have both reflected and reproduced broader tensions around equity, governance, and quality in a multilingual and post-conflict society.

Following the country’s independence in 1991, the higher education sector remained relatively limited in size and scope, with Macedonian as the dominant language of instruction and limited provision for minority groups. However, the 2001 OFA represented a decisive turning point by institutionalising multilingualism and decentralised governance in education, laying the legal and political foundations for minority-language instruction and expanded access to higher education (OECD Report, 2004; Pollozhani, 2022).

To situate readers unfamiliar with the North Macedonian context, Table 1 provides a structured overview of the higher education landscape. According to the State Statistical Office (2024), a total of 52,316 students were enrolled across North Macedonian higher education institutions in the academic year 2023/2024 with 77.9% enrolled in public institutions and 22.1% in private ones. The gross tertiary enrolment rate stands at approximately 55% (World Bank, 2023). The higher education system includes public universities, private universities, one public-private institution, and other providers such as professional higher education institutions, scientific institutes, and a foreign higher education institution; all are subject to national accreditation and quality assurance requirements.

**Table 1:** Overview of higher education institutions in North Macedonia (2024/2025)

Institution	Status	Primary language(s) of instruction	EMI status	Degree levels
Ss. Cyril and Methodius University (Skopje)	Public	Macedonian, Albanian	Selected programmes/courses	BA, MA, PhD
St. Clement of Ohrid University (Bitola)	Public	Macedonian	Limited EMI	BA, MA, PhD
Goce Delchev University (Štip)	Public	Macedonian	Limited EMI	BA, MA, PhD
State University of Tetovo	Public	Albanian, Macedonian	Limited EMI	BA, MA, PhD
Mother Teresa University (Skopje)	Public	Albanian, Macedonian	Partial (Informatics only)	BA, MA, PhD
University of Information Science and Technology “St. Paul the Apostle” (Ohrid)	Public	English	Full EMI – all programmes	BA, MA, PhD
South East European University – SEEU (Tetovo)	Public-private (international)	Albanian, Macedonian, English	Extensive EMI – most programmes	BA, MA, PhD
University American College Skopje	Private	English, Macedonian	Extensive EMI	BA, MA
International Balkan University (Skopje)	Private	English, Turkish, Macedonian	Extensive EMI	BA, MA
European University (Skopje)	Private	Macedonian, English	Partial EMI	BA, MA
FON University (Skopje)	Private	Macedonian	Limited EMI	BA, MA
International Slavic University “G. R. Derzhavin”	Private	Macedonian	Limited EMI	BA, MA
International University of Struga	Private	Albanian, Macedonian	Limited EMI	BA, MA
Euro-Balkan University (Skopje)	Private	Macedonian	Limited EMI	BA, MA
International Vision University (Gostivar)	Private	Albanian, English, Turkish	Partial EMI	BA, MA
MIT University (Skopje)	Private	Macedonian, English	Partial EMI	BA, MA
University of Tourism and Management (Skopje)	Private	Macedonian	Limited EMI	BA, MA
University for Audiovisual Arts – ESRA (Skopje)	Private	French, Macedonian	No/minimal EMI	BA
University of Studies Struga “EuroCollege”	Private	Albanian, Macedonian	Limited EMI	BA, MA
Military Academy “General Mihailo Apostolski” (Skopje)	Public	Macedonian, English	Partial EMI	BA, MA, PhD
New York University Skopje (NYUS)	Private	Macedonian, English	Extensive EMI	BA, MA

Total enrolment 52,316 students (2023/24); ~78% in public institutions, ~ 22% in private institutions (State Statistical Office, 2024). Gross tertiary enrolment rate ~ 55% (World Bank, 2023). EMI classification is based on publicly available institutional websites and the analysis presented in this study. “Full EMI” = all programmes delivered in English; “Extensive EMI” = majority of programmes include English-medium options; “Partial EMI” = some programmes or selected courses in English; “Limited EMI” = only isolated courses or no systematic EMI

The list is not exhaustive as various official sources have different numbers because some of the focus on higher education institutions whereas some others on universities

A major shift occurred in the mid-2000s, characterised by the intensification of higher education reforms aligned with the Bologna Process (Galevski, 2021). The adoption of European Credit Transfer and Accumulation System (ECTS) standards, the creation of national quality assurance bodies, and the implementation of student mobility frameworks signalled a move toward alignment with the European Higher Education Area (Zhivkovikj, 2019). These reforms coincided with the liberalisation of the higher education market, leading to the rapid multiplication of both public and private institutions and the proliferation of new study programmes (Spasovski & Pecakovska, 2020).

As of 2025, North Macedonia hosts 21 higher education institutions, comprising seven public universities and 14 private ones (European Commission/EACEA/Eurydice (2026). While public institutions such as Ss. Cyril and Methodius University in Skopje, St. Clement of Ohrid University in Bitola, and Goce Delchev University in Štip remain central to the higher education system; private providers have significantly expanded, often with diverse linguistic and institutional models. Some of these, such as the University American College Skopje, International Balkan University and the South East European University (SEEU), offer instruction in English, Macedonian, Albanian, and Turkish (Bigagli, 2021; Koksal, 2014; Tomovska Misoska et al., 2024).

However, this expansion has not been without challenges. The rise of private institutions, enabled in part by government licensing mechanisms, has triggered debates around the monetisation of education and the commodification of academic credentials (Spasovski & Pecakovska, 2020). In many cases, private universities have predominantly catered to students from affluent backgrounds, raising concerns about equity, academic standards, and the decline in diversity within student bodies (Krampf & Heinlein, 2014; Ferguson et al., 2018; Sokoli et al., 2025). Simultaneously, the rapid growth of institutions has led to increased demand for qualified faculty, exacerbating shortages across both public and private sectors (Sokoli et al., 2025).

Another issue that characterises the North Macedonian higher education system is the ethnification of education (Stojanovski et al., 2018). Segregation based on ethnicity and language remains visible across all levels of education, including universities. This has contributed to the development of parallel educational structures, particularly between Macedonian and Albanian communities, which scholars argue impedes cross-cultural dialogue and undermines long-term goals of social cohesion and reconciliation (Czaplinski, 2008; Galevski, 2021).

In sum, the North Macedonian higher education system is characterised by institutional diversification, linguistic plurality, and persistent challenges in quality and equity. The interplay of liberalisation, Europeanisation, and post-conflict reconciliation has produced a complex terrain where language, governance, and market forces intersect. Within this landscape, EMI functions as both a strategic institutional response and a by-product of macro-level reforms, raising critical questions about access, quality, and the role of higher education in a multilingual society.

### EMI in the North Macedonian higher education

Research on EMI in North Macedonia remains sparse (Gavriilidou & Mitits, 2023), with only a few studies, such as Agai-Lochi (2015), examining EMI programmes. Nevertheless, EMI has expanded considerably in recent years. All six public universities in the North Macedonian higher education system now offer at least some courses in English, although their scope and institutional integration vary. For instance, according to their websites, Mother Teresa University offers EMI only in Informatics, while the University of Information Science and Technology *St. Paul the Apostle* offers all programmes in English. Among private institutions, EMI is even more prevalent, with some universities offering only English-taught programmes and others operating in multiple languages, including Macedonian, Albanian, Turkish, and English.

The growth of EMI is often linked to the perceived prestige of English, concerns about employability, and a drive toward internationalisation. As highlighted in strategic documents, universities increasingly frame EMI as a means of aligning with European labour market demands and enhancing North Macedonia’s competitiveness in attracting foreign investment and students (SEEU, 2024). However, this expansion has occurred in the absence of a clearly defined national EMI policy, with regulatory frameworks and institutional strategies evolving in an ad hoc manner.

It can be argued that the rise of EMI has emerged as both a response to and a reflection of broader transformations. Institutions such as SEEU and the University of Information Science and Technology *St. Paul the Apostle* in Ohrid have pioneered EMI offerings, explicitly linking them to the goals of modernisation, internationalisation, and employability. However, this has occurred largely in the absence of a national EMI policy, reflecting a pattern of decentralised, institution-driven initiatives aligned with liberalised governance models. While demand and institutional initiative play an important role, the macro-level drivers of EMI in North Macedonia—such as state reforms, quality assurance pressures, and international donor involvement—have yet to be systematically examined.

The governance-oriented approach adopted in this study complements, rather than replaces, the insights generated by critical language policy studies (CLPS). Scholars working in this tradition, including Tollefson (1991, 2006), Phillipson (1992), and Ricento (2000), have established that choices about medium of instruction are never linguistically or educationally neutral but are embedded in broader structures of power, ideological reproduction, and social inequality. These insights are foundational to understanding why English occupies the position it does in global higher education. However, CLPS scholarship has tended to focus on the ideological dimensions of language policy and the intentional reproduction of linguistic hierarchies, whereas the North Macedonian case presents a distinct analytical challenge: here, English did not gain institutional prominence through deliberate ideological promotion or explicit colonial or neo-imperial imposition, but as an unintended by-product of governance reforms oriented toward entirely different objectives. It is precisely this gap between ideological intent and structural outcome that the concept of policy spillover, operationalised through SPT, is designed to address. Our approach therefore extends the CLPS tradition by showing that power-laden language shifts can be produced not only through deliberate ideological management but also through the cumulative, unintended effects of governance reforms that make no explicit reference to language at all.

### Steering at a distance

The concept of steering at a distance, introduced by Kickert (1995), may offer a lens for understanding how North Macedonia’s higher education reforms have shaped the EMI landscape. Under this governance model, states retreat from direct control but maintain influence through accountability measures, funding mechanisms, and strategic frameworks (Hultgren et al., 2023; Bleiklie, 2018). These mechanisms include performance-based funding, institutional evaluations, and development contracts, all of which have been progressively introduced in the North Macedonian higher education sector since the early 2000s.

A critical conceptual challenge in analysing EMI within governance frameworks such as steering at a distance concerns the analytical utility of the distinction between top-down and bottom-up language policy. In the EMI literature, top-down policy is conventionally associated with deliberate, state-initiated language planning. This policy explicitly mandates that designate English as a medium of instruction at the national level, while bottom-up emergence is attributed to institutional or grassroots actors responding to local conditions (Kaplan & Baldauf, 1997; Spolsky, 2004). However, this binary has been increasingly problematised. Spolsky, (2004, 2009) argues that language policy should be understood as comprising three interconnected components, i.e.,language practices, language beliefs or ideologies, and language management. The forces shaping these components may be non-linguistic in origin, operating from within or outside the relevant domain. Critically, this means that governance reforms ostensibly unrelated to language can function as de facto language management, producing language shifts without any explicit linguistic intent. Shohamy (2006) extends this argument further, distinguishing between overt, declared language policies and covert mechanisms, what she terms policy devices, through which ideologies are translated into practices without formal linguistic declaration. These devices include curricula, accountability frameworks, and institutional incentive structures that, while not constituting language policy in name, exert profound influence on language use in practice.

This theoretical lens is particularly productive for contexts where EMI appears to emerge without explicit top-down mandates. Comparative evidence from European higher education systems suggests that EMI expansion, even where it resembles deliberate planning, is frequently reactive to deeper structural shifts rather than the product of coherent linguistic intent. Airey et al., (2017) demonstrate this in their analysis of four Nordic countries, finding that institutions have generally adopted English with little systematic reflection about where it may or may not be appropriate, driven instead by Bologna-related internationalisation pressures and the logic of institutional prestige. A directly comparable case is provided by the Turkish higher education system, where process tracing analysis reveals that the growth of EMI programmes was driven primarily by three structural milestones in that country: the establishment of the Council of Higher Education, massification, and privatisation efforts, none of which constituted explicit language policy, yet which collectively created the conditions for EMI to flourish (Yuksel et al., 2025). Together, these cases suggest that the apparent presence or absence of explicit top-down language planning may be less analytically significant than the structural conditions—governance reforms, marketisation, supranational pressures—that produce language shifts as unintended or covertly engineered by-products.

This insight is central to the present study’s contribution. Rather than positioning North Macedonia’s EMI landscape as either a failure of top-down planning or a product of bottom-up institutional entrepreneurship, we argue that this binary is itself inadequate for capturing how language change operates within post-socialist governance systems shaped by steering at a distance. Drawing on Shohamy’s (2006) concept of covert policy mechanisms and Spolsky’s, (2004, 2009) understanding of non-linguistic forces as drivers of language management, we propose that the concept of policy spillover captures a distinct causal pathway: one in which reforms designed for political, economic, or social objectives inadvertently produce language shifts as institutionalised by-products. This framing relocates the locus of analysis from the intent of policymakers to the structural conditions and interpretive processes through which language change is produced, a move that, we argue, is both theoretically more precise and empirically more adequate for contexts where EMI has expanded in the absence of explicit language planning.

In this context, universities are expected to operate autonomously while aligning with national and European objectives, including internationalisation and labour-market relevance. As such, EMI has emerged not only as a linguistic or instructional development but also as a response to broader governance reforms that reward institutions for adaptability, competitiveness, and global visibility (Xhihani, 2025; Stojanovski et al., 2018). The growth of EMI in North Macedonia can therefore be understood as a case of policy spillover — an institutionalised by-product of steering-at-a-distance governance, particularly in a system where international aid and European integration agendas play an outsized role in shaping institutional strategies.

Despite the growing presence of EMI in North Macedonian higher education, there is a lack of systematic research exploring how governance reforms and macro-level language policy shape this trend. Most existing accounts offer descriptive overviews or focus on institutional motivations, leaving unexamined the broader political and structural forces that enable or constrain the rise of EMI (e.g., Agai-Lochi, 2015). In particular, little attention has been paid to the interplay between language policy, decentralised governance, internationalisation pressures, and the legacies of post-conflict educational restructuring.

This study aims to determine how state-level mechanisms—specifically the intersection of higher education governance reform and language policy—have shaped the trajectory of EMI. The research focuses on identifying the specific regulatory and political forces that have transformed the Macedonian higher education landscape to accommodate English alongside dominant and minority languages.

## Theory and method

This study forms part of a broader research agenda that seeks to understand the mechanisms driving the rise of EMI across diverse European higher education contexts as a part of the UKRI-funded Elemental project (e.g., Nao et al., 2023; Nao et al., 2025; Thomas et al., 2024; Yuksel et al, 2025; Zuaro et al., 2024). Within this scope, North Macedonia offers a particularly compelling case due to its complex multilingual landscape, post-conflict political environment, and ongoing reforms in higher education governance. Adopting a transdisciplinary perspective, the present study integrates insights from political science, language policy and planning (LPP), and higher education studies to examine how EMI emerged and evolved in a context shaped by both decentralisation and internationalisation pressures.

The conceptual framework of this research integrates two interrelated theoretical constructs: steering at a distance and policy spillover. Steering at a distance, first theorised by Kickert, (1995) in the context of Dutch higher education governance, describes a mode of state control in which governments withdraw from direct management of public institutions while retaining influence through indirect mechanisms such as performance-based funding, quality assurance systems, accreditation frameworks, and strategic planning requirements (Bleiklie, 2018; Chatelain-Ponroy et al., 2018; Lingard, 1996; Marginson, 1997). The core theoretical assumption of this model is that institutional autonomy is not a withdrawal of state power but rather a reconfiguration of it: by setting the rules of the competitive field rather than prescribing specific outcomes, the state shapes institutional behaviour at a distance. Crucially, this means that institutions internalise external expectations, including internationalisation targets or labour market alignment, and act on them proactively, without requiring explicit directives. In the context of EMI research, steering at a distance provides a framework for understanding why universities adopt English-medium programmes not because they are told to, but because the governance environment structurally rewards them for doing so (Zuaro et al., 2025).

The second construct, policy spillover, derives from the political science literature on unintended policy consequences (Barzelay & Gallego, 2006) and describes a process whereby policies designed to achieve objectives in one domain produce significant, unanticipated effects in another. In the present study, we apply this concept to the language policy domain, arguing that successive higher education governance reforms in North Macedonia, which aimed at ethnic inclusion, privatisation, Bologna harmonisation, and institutional liberalisation, functionally operated as de facto language policies, producing the conditions for EMI to emerge and consolidate even in the absence of any explicit national language planning mandate. Taken together, steering at a distance and policy spillover are not merely descriptive labels but constitute the causal logic of our analytical framework: the state steers institutions through structural incentives (steering at a distance), and those incentives, operating across multiple reform domains simultaneously, produce language change as a cumulative, unintended by-product (policy spillover).

### Social process tracing

In this study, we employ SPT, a methodological variant of process tracing particularly suited to analysing complex policy environments in which human interpretation plays a central role. While traditional process tracing often focuses on establishing positivist causal chains (Beach & Pedersen, 2019), SPT adopts an interpretivist stance, encompassing “both the moves performed by actors, the meanings they ascribe to them, and how the underlying social context shapes how social causal processes play out” (Kaas et al., 2025: 605). This approach aligns with the core concerns of applied linguistics by placing the social context and the actors’ meaning-making processes at the heart of the inquiry (Zuaro et al., 2025).

We utilised an iterative, abductive research design (Schwartz-Shea & Yanow, 2013; Tavory & Timmermans, 2014) to reconstruct the rise of EMI systematically. Rather than testing a pre-determined hypothesis, this involved moving back and forth between our theoretical framework of steering at a distance and the empirical data. This process allowed us to identify key episodes—crucial moments of interaction where we had theoretical reasons to expect that the process might have played out differently if different actions had been taken (Steel, 2007). By focusing on these episodes, we were able to trace how macro-level governance reforms (the cause) were interpreted by university leaders and policymakers (the actors), eventually leading to the institutionalisation of English-taught programmes (the outcome). This highlights that the researcher is a key instrument in understanding the meanings actors ascribe to their actions (Fujii, 2017), allowing us to uncover how non-linguistic policies were translated into de facto language planning.

### Data sources and participants

This study draws on two primary sources of data: a documentary analysis of relevant legal, policy, and institutional texts, and semi-structured interviews with key stakeholders involved in higher education governance and administration in North Macedonia (see Appendix for the list of questions). The documentary corpus included national legislation related to higher education and language-in-education policy, such as the Law on Higher Education, the Skopje City Law, and legal provisions introduced following the signing of the Ohrid Framework Agreement. It also comprised strategic policy documents, notably the 2005 National Strategy for the Development of Education, as well as institutional-level materials including university mission statements, development plans, accreditation guidelines, and quality assurance frameworks from both public and private universities.

The interviews were conducted with 10 elite participants—individuals occupying influential positions in higher education governance or within university administration. These included former and current rectors and vice-rectors, faculty deans, department heads of North Macedonian universities, and senior officials from the Ministry of Education and Science. Their selection was guided by prior document analysis, which identified institutional actors involved in the creation or implementation of EMI programmes. Participants were selected using purposive sampling, with snowballing used to expand the sample. Interviews were conducted in Turkish or English, reflecting both the linguistic competences available to the researcher conducting the data collection and the preferences expressed by participants, who were offered a choice between these two languages and opted accordingly. While Macedonian and Albanian are the dominant languages of the national context, neither fell within the shared communicative repertoire of both the researcher and all participants. All interviews were conducted with informed consent, aiming to capture policy rationales, institutional strategies, and historical developments from the perspective of decision-makers. Basic information regarding the elite stakeholders is provided on Table 2.

**Table 2:** Basic information on interview participants

Participant	Role	Institutional type	Language of interview	Approximate duration
P1	Former department head	Private university	Turkish	45 min
P2	Former rector	Public university	English	60 min
P3	Former vice-rector	Public university	Turkish	54 min
P4	Former vice rector	Private university	English	59 min
P5	Faculty dean	Public university	Turkish	55 min
P6	Former vice-rector	Public university	English	64 min
P7	Department head	Private university	Turkish	5 min
P8	Senior official	Ministry of Education and Science	English	67 min
P9	Vice-rector	Public university	English	59 min
P10	Former rector	Private university	Turkish	63 min

All participants are anonymised. Role descriptions reflect positions held at the time of the study or at the time of the relevant policy events discussed

### Data collection and analysis procedures

The study began with a comprehensive analysis of legal and policy documents to establish a timeline of reforms related to language use and EMI in higher education. Key events were mapped, including the 1991 Constitution, the 2001 Ohrid Framework Agreement, the founding of South East European University, and Macedonia’s participation in the Bologna Process (see Table 3 for further details). This document analysis enabled the identification of turning points in the governance of multilingual and English-medium higher education, paving the way for the SPT analysis.

Interview data were collected and analysed thematically. The questions were tailored to elicit narratives about institutional responses to policy changes, perceptions of EMI’s value and function, and the role of international actors in shaping educational reforms. Interview transcripts were coded iteratively, with a focus on identifying causal mechanisms and actor roles in policy processes. In line with SPT conventions, the quotes selected for presentation in the findings represent the most analytically precise articulation of interpretive patterns identified across multiple participants and corroborated by documentary sources, rather than constituting the sole evidential basis for the claims made. For instance, responses from former rectors and ministry officials highlighted how the implementation of the Bologna Process and the pressures of EU integration influenced universities to adopt EMI as part of their internationalisation strategies. Similarly, documents and interviews highlighted the role of foreign donors in establishing institutions such as SEEU and promoting the use of English as a medium of instruction.

Data triangulation was employed to enhance the validity of the causal claims produced through SPT. Specifically, three types of cross-referencing were conducted. First, participants’ accounts of specific policy events and institutional decisions were checked against contemporaneous legal documents, ministerial decrees, and higher education laws to assess whether retrospective interview narratives were consistent with the documentary record at the time of the reforms in question. Second, institutional actors’ descriptions of their motivations for adopting EMI were cross-referenced with university strategy documents, mission statements, and accreditation frameworks produced by their own institutions, allowing us to assess the alignment between stated rationales and formally documented institutional positions. Third, accounts offered by participants from different institutional positions, namely public university administrators, private university leaders, and ministry officials, were compared with each other to identify convergent patterns of interpretation as well as points of divergence that required further analytical attention. Where interview accounts and documentary sources aligned, this convergence was treated as strengthening the evidential basis for the causal claims made. Where discrepancies arose, these were noted and examined as analytically significant in their own right, reflecting the SPT principle that actors’ interpretations of governance signals, rather than the signals themselves, constitute the primary causal mechanism under investigation.

While this study is grounded in the specific socio-political context of North Macedonia, we do not view our findings as purely idiosyncratic. In line with SPT, we aim to construct a social process theory that is particularised to this case yet offers analytical generalisability to broader contexts (Kaas et al., 2025). By identifying the specific causal mechanisms through which EMI emerged—traced through policy shifts, actor meanings, and key episodes—we provide a theoretical framework that can be assessed in other settings. This approach allows the identification of functional equivalence at the process level (Kaas et al., 2025), suggesting that the dynamics of policy spillover and steering at a distance observed here may offer explanatory power for other post-socialist or transitional higher education systems navigating similar governance reforms.

## Findings

This section presents the findings from our SPT analysis of how English-taught programmes emerged and proliferated in North Macedonia’s higher education sector. Drawing on legal and policy documents, university strategies, and elite interviews, we identify five key episodes that, over time, enabled EMI to become embedded in institutional practices. While we present these episodes in chronological order, it is crucial to clarify that their relationship is causal rather than merely sequential. We trace a generative process where the specific outcomes and social context established in one phase created the necessary conditions for the next. This allows us to demonstrate how the trajectory evolved from post-conflict multilingual accommodation to strategic marketisation, revealing the causal chain through which distinct governance reforms cumulatively produced the policy spillover of EMI.

### Post-independence nation-building and marginalisation of linguistic minorities (1991–2000)

The early post-independence period was marked by systemic exclusion of minority languages in higher education, reflecting nation-building strategies. The 1991 Constitution downgraded the political and linguistic status of ethnic Albanians, contributing to educational disenfranchisement. For instance, the closure of the unrecognised Albanian-language University of Tetovo by government forces in 1995 became a flashpoint in this exclusionary policy landscape. This, however, prompted backlash from Albanian-speaking communities. As one former vice-rector explained:

*The lack of tertiary-level education in Albanian was a major concern among many Albanians living in Macedonia. Most people in Albanian-speaking communities believed that the educational system simply did not reflect the country’s multilingual and multi-ethnic realities. This directly impacted many Albanians as universities were gateways to jobs and prestige.* (Participant 3, former vice-rector of a state university)

This assessment was consistent across participants who had experienced or administered higher education during this period, and is corroborated by the documentary record: the 1991 Constitution, the 1997 Law on Languages, and the 2000 Higher Education Law collectively trace the legal trajectory of exclusion and incremental accommodation that participants described. In response to such exclusionary policies, ethnic minorities began to mobilise, and incremental legal changes emerged. The 1997 Law on Languages permitted minority-language tuition at pedagogical faculties, and by 2000, minority-language provision was incorporated into the Law on Higher Education, laying the legal groundwork for institutional multilingualism and, indirectly, for the later accommodation of English. Critically, this legal normalisation of linguistic plurality within higher education was a necessary precondition for what followed: without it, the post-conflict settlements of Episode 2 would have lacked the institutional and legal scaffolding needed to establish a multilingual, and in some cases English-medium, university model with state recognition.

### Conflict, the Ohrid framework agreement, and multilingual settlements (2001–2004)

The 2001 armed conflict between ethnic Albanian insurgents and state forces catalysed structural change in higher education governance and language rights. The Ohrid Framework Agreement (OFA) institutionalised multilingualism and mandated the use of minority languages in education where spoken by at least 20% of the population. This period saw the founding of South East European University (SEEU)—a landmark institution designed to embody conflict resolution through multilingual education. SEEU was also the first institution in the country to explicitly embed EMI within its trilingual model. As a founding administrator of SEEU explained:

*A conflict-resolution case… it was designed not only to offer education in Albanian but to show that multilingualism can work within a unified institutional framework. We were very excited to be a part of this change in the higher education system. (Participant 1, former department head of a private university)*

This characterisation of SEEU as a conflict-resolution vehicle was not unique to this participant; it was consistently present across interviews with administrators from both public and private institutions who had witnessed or participated in the post-OFA restructuring of higher education, and is corroborated by the OECD Report (2004) and the institutional documentation of SEEU’s founding mandate. The international support behind SEEU, particularly from EU and US donors, signalled the beginning of external involvement in shaping North Macedonia’s higher education architecture, embedding English, among other languages, into a post-conflict peacebuilding framework. According to its website, SEEU is seen “as a model for multi-ethnic, multilingual higher education in South East Europe” (SEEU, 2024: n.p). In doing so, Episode 2 produced three institutional conditions upon which subsequent EMI expansion critically depended: a legitimised precedent for English as an academic language within a state-recognised institution; an embedded network of international donor relationships that would continue to shape institutional incentives; and a decentralised governance architecture that granted universities the autonomy to develop their own linguistic models without requiring central ministerial approval.

### Europeanisation and systemic governance reform (2004–2009)

Following its 2004 EU membership application, North Macedonia intensified governance reforms to align with the Bologna Process. This phase was characterised by performance-based governance tools, decentralisation, the establishment of quality assurance mechanisms, and the adoption of the European Credit Transfer and Accumulation System (ECTS). These reforms created a policy environment conducive to institutional innovation, including the rise of English-taught programmes. As one policymaker remarked:

*A gateway not only to harmonisation but to competitiveness—we had to be seen as moving closer to the European Higher Education Area*,* and English became a key part of that story. We believed that if we offered programmes in English*,* we could more easily integrate the Bologna Process and ECTS*. (Participant 6, former vice-rector of a public university)

This trend was materialised in 2009 with the foundation of a public university in Ohrid, the University of Information Science and Technology *St. Paul the Apostle*, which launched all its programmes exclusively in English. Here, English was positioned not merely as a medium of instruction, but as a tool for global market integration, signalling institutional modernity and EU alignment. Episode 3 thus transformed EMI from a conflict-resolution accommodation, as it had functioned in Episode 2, into a governance-aligned institutional strategy, establishing the symbolic and regulatory conditions under which private universities, emerging under marketisation in Episode 4, would adopt EMI not as a peace-building tool but as a competitive market instrument. With international students from more than 40 countries, the University of Information Science and Technology is the first public university in North Macedonia which offers all programmes in English.

### Marketisation and strategic positioning (2010–present)

From 2010 onwards, the discourse around EMI in North Macedonia began to shift significantly. No longer confined to post-conflict multilingual accommodation, EMI was increasingly framed by universities—particularly private institutions—as a strategic response to the pressures of globalisation, economic restructuring, and international competitiveness. Strategic documents from both public and private institutions began to explicitly link EMI to labour market alignment, foreign direct investment, and institutional modernisation. This phase reflects a broader transition from peacebuilding logics to neoliberal rationales in higher education policy.

One clear illustration of this discursive turn can be found in the strategy documents of the SEEU, which present EMI not merely as an academic tool, but as an economic instrument:

*[SEEU offers] teaching and research in English as an international language of communication in most of our fields of study… [and] aims to diversify its academic programmes further to enable graduates to pursue new opportunities in a rapidly-changing employment market.* (SEEU Strategic Plan for 2021–2025)

Such strategic framings were not limited to mission statements; they were echoed across our interviews with institutional leaders. Several participants described teaching in English as a powerful institutional signifier, projecting values of innovation, internationalism, and prestige. As one rector from a private university noted:

*There’s a symbolic value in offering a programme in English—it tells the student*,* and the outside world*,* that we are global*,* forward-looking*,* and competitive. It also directly opens our programmes to international students. Those coming from neighbouring states*,* as well as those who shared historical backgrounds with us*. (Participant 4, former rector of a private university)

While this quote is drawn from a private university administrator, the symbolic association of English with institutional modernity and global competitiveness was not confined to private sector actors. Participants from public universities and the Ministry of Education and Science similarly described English-medium provision as carrying reputational value, though they framed it more in terms of EU alignment than market differentiation. This symbolic function of EMI was especially pronounced within private universities, many of which had been established in the context of market liberalisation. These institutions were quick to seize on EMI as a mechanism of differentiation in an increasingly saturated higher education market. The growth of EMI in these contexts was closely tied to the broader trend of privatisation, which was itself enabled by governance reforms that loosened state control over accreditation and programme development.

*The rise in private universities*,* often with a significant number of programmes in English*,* is a direct response to a perceived demand that the public system was not fully doing what they were supposed to be doing. We are just offering more appealing*,* somehow specialised programmes.* (Participant 4, former rector of a private university)

What emerges in this period is a convergence of symbolic and structural incentives: universities adopted EMI both to position themselves within international higher education networks and to respond to evolving regulatory regimes that rewarded institutional and academic autonomy, innovation, and global alignment. EMI thus became embedded not through a singular national directive, but through institutional strategic repositioning within an environment increasingly shaped by market logics and international benchmarks.

### Intersecting internal and external drivers of EMI growth

While the previous section traced the evolution of institutional discourses around EMI in the post-2010 period, this section unpacks the drivers of that transformation. Our data reveal that the rise of EMI in North Macedonia was not the product of a single reform, actor, or directive. Rather, it was the result of a multi-layered process involving the intersection of external pressures, internal institutional adaptations, and macro-political change.

Externally, EMI was driven by Europeanisation agendas, the Bologna Process, international aid programmes, and the reputational pressures of global university rankings. These factors collectively pushed institutions to align their structures and offerings with perceived global standards. For instance, as one senior policymaker in the Ministry of Education and Science noted:

*The prospect of EU integration acts as a powerful motivator… There’s a clear understanding that our educational system needs to be a facilitator*,* not an impediment*,* to this integration. We observe many changes in our governance system*,* and educational governance also supports our EU integration agenda* (Participant 8, a policymaker in the Ministry of Education and Science).

International development agencies, particularly during the post-conflict period, were instrumental in laying the groundwork for EMI, not just through funding but through technical expertise and institutional partnerships. As one former vice-rector from a private EMI university reflected:

*International aid has played a significant role*,* especially in the early stages of our transition… However*,* we need to be mindful that aid should foster sustainable development.* (Participant 1, former department head of a private university)

Additionally, global ranking systems and accreditation frameworks exerted subtle yet sustained pressure on universities to adopt policies associated with internationalisation, including EMI. The perceived correlation between English-language programmes and global academic status was a recurring theme:

*University rankings*,* while sometimes controversial*,* exert an undeniable influence… They’ve pushed our institutions to focus on research output*,* international collaboration*,* and visibility. When we meet our potential students*,* rankings is always on the table as a driving force of recruitment for us. Our students always want to learn more about our position compared to other private universities in our country.* (Participant 4, former rector of a private university)

Crucially, EMI was not only viewed as a functional practice but also as a reputational and symbolic asset for some university administrators. For institutions seeking visibility, especially in competitive urban centres, English-language offerings became a proxy for academic quality and global relevance:

*Using English… is instrumental. We want to attract international students and to prepare our local students for a globalised world… There’s a perceived ‘prestige’ associated with programmes in English*,* both for domestic and international students.* (Participant 4, former rector of a private university)

These dynamics align closely with the concept of steering at a distance: while the state did not directly mandate EMI, it created a governance environment—through quality assurance agencies and policy frameworks—that rewarded alignment with international norms. Universities, operating with increased autonomy but within a competitive, metrics-driven landscape, began to view EMI as both a compliance strategy and an institutional opportunity.

These findings are further corroborated by the policy timeline and Table 3, which show a consistent pattern: while no national policy explicitly mandated EMI, successive reforms—whether aimed at multilingual inclusion, privatisation, marketisation, Bologna harmonisation, or institutional liberalisation—contributed to the conditions under which EMI could thrive. This cumulative, indirect process—what we term policy spillover—demonstrates how EMI became institutionalised as a by-product of broader governance transformations.

**Table 3:** Review of macro-level language policy actions in North Macedonia and their focus and impact

Year	Action	Focus	Impact	Source
1991	The new Constitution was adopted after independence	Ethnic and linguistic minority status in higher education governance	Negative	Ilievski (2007); )Škaric (2004)
1995	The government forcibly shuts down the unrecognised Tetovo University	Access to higher education in minority languages	Negative	Ilievski (2007);
1997	The Law on Languages permits tuition in minority languages at the Pedagogical Faculty.	Language rights in higher education	Positive	Bliznakovski (2013)
2000	Minority language provision incorporated into the Higher Education Law	Legal foundation for multilingualism	Positive	Bliznakovski (2013)
2001	Ohrid Framework Agreement signed	Multilingualism, decentralisation, positive discrimination	Positive	Škaric (2004); OECD (2004)
2001	Establishment of South East European University (SEEU) with EMI component	Conflict resolution, internationalisation, multilingual model	Positive	Ilievski (2007); SEEU documents
2003	Tetovo University is formally recognised as a state institution	Inclusion of Albanian-language HEI	Positive	OECD (2004)
2003	The new Higher Education Law allows instruction in minority languages in state universities	Linguistic equality and access	Positive	OECD (2004)
2004	Macedonia applies for EU membership	Europeanisation of education	Positive	Policy timeline
2005	National Strategy for the Development of Education adopted	Internationalisation, mobility, and reform	Positive	Kreceva (2008)
2009	The University of Information Science and Technology opens with full EMI provision	Promotion of English as a medium in specialised HE	Positive	Institutional documents
Post-2010	University strategy documents promote EMI for competitiveness and employability	Language policy as a tool for marketisation	Positive	SEEU strategy; Interviews

Impact ratings reflect each policy action’s assessed effect on the conditions enabling linguistic pluralism and English-medium provision in North Macedonian higher education, as evaluated through documentary analysis and elite interview data collected for this study. A Positive rating indicates that the action expanded linguistic access, institutionalised multilingualism, or created governance conditions conducive to EMI development. A Negative rating indicates that the action restricted minority language rights, limited access to higher education for linguistic minorities, or created governance conditions that constrained linguistic pluralism

### Synthesis: EMI as a causally sequenced governance outcome

**Fig. 1:**
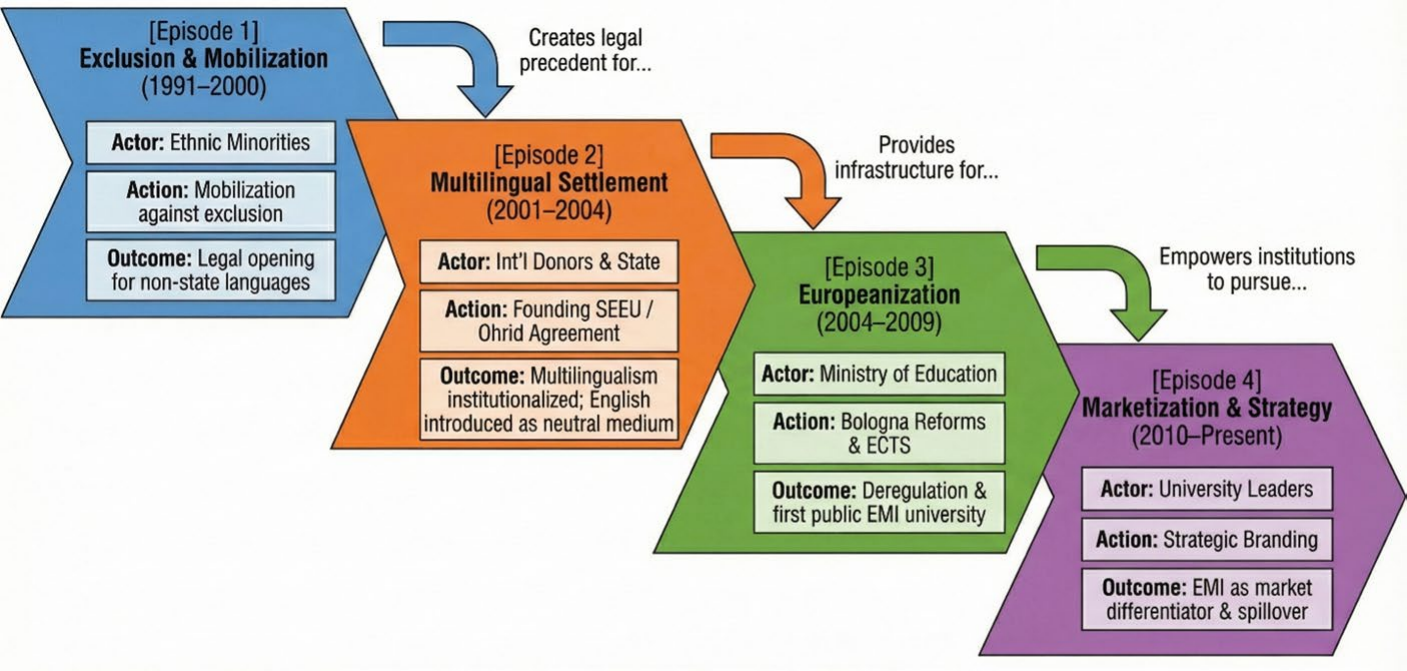
A causal social process tracing analysis of EMI growth in North Macedonia. *Note*: The figure illustrates the generative mechanism in which outcomes in one phase (e.g., post-conflict multilingualism) serve as necessary conditions for subsequent reforms (e.g., Europeanization), cumulatively resulting in the spillover of EMI

Across these five phases, EMI in North Macedonia evolved from a marginal feature of peacebuilding efforts to a central strategy for institutional differentiation and internationalisation, as illustrated in Fig. 1. Crucially, this trajectory was not merely chronological but causally generative: the outcomes and institutional conditions produced in each episode functioned as necessary preconditions for the mechanisms that drove EMI in the next.

This causal sequencing is what distinguishes the North Macedonian pathway from contexts where EMI has been adopted without analogous early stages. In many European settings, particularly Western and Northern European systems, EMI expansion has been driven primarily by Bologna-era internationalisation pressures and market competition, without a prior history of post-conflict multilingual accommodation (Airey et al., 2017; Galloway et al., 2020). In North Macedonia, by contrast, EMI could not have acquired institutional legitimacy through the Bologna process alone, because the pre-existing governance environment did not recognise linguistic plurality in higher education. It was the post-independence legal reforms of Episode 1 that first established the institutional principle of non-Macedonian instruction; and it was the post-conflict settlements of Episode 2 that embedded English specifically as a legitimate academic medium, normalised external donor involvement in shaping institutional models, and introduced the decentralised governance architecture that gave universities the autonomy to develop distinct linguistic strategies. Without these prior conditions, the Europeanisation pressures of Episode 3 would have encountered a governance environment far less receptive to EMI adoption.

Understood in this way, the causal argument is not that post-conflict multilingualism inevitably produces EMI, and the North Macedonian case does not make that claim. Rather, the argument is that in a post-socialist, post-conflict, multilingual society navigating simultaneous pressures of ethnic reconciliation, EU accession, and market liberalisation, the specific sequencing of these reforms created a unique confluence of institutional legitimacy, governance decentralisation, donor networks, and competitive incentives that made EMI the path of least resistance for institutions seeking to signal compliance, modernity, and international alignment. The policy spillover of EMI was, in other words, the product of a conjunctural alignment of these conditions, and it is the specific historical and political configuration of North Macedonia that explains both why EMI emerged and why it took the particular institutional form it did.

## Discussion

This study has examined the rise of EMI in North Macedonia not as a stand-alone language policy initiative, but as the cumulative policy spillover of overlapping governance reforms. Through an SPT methodology grounded in the theory of steering at a distance (Kickert, 1995), our analysis reveals a more critical insight than the simple absence of national planning. By focusing on the social meanings actors ascribed to governance reforms, we found that the proliferation of EMI was largely decoupled from pedagogical or educational motivations. Instead, it emerged as a strategic currency for institutional actors navigating a marketised landscape; university leaders adopted English primarily to signal compliance with European standards and to accrue institutional prestige. Thus, our SPT reveals that EMI in this context mainly functions as a compliance mechanism and a branding tool and marketing tool, rather than as the product of a coherent linguistic or educational strategy.

### EMI as a product of multilevel governance transformation

The trajectory of EMI in North Macedonia mirrors the broader restructuring of its higher education landscape since the early 1990s (Spasovski & Pecakovska, 2020). From the early post-independence marginalisation of minority languages to the peacebuilding goals of the Ohrid Framework Agreement (2001), language policy initially focused on internal cohesion and minority inclusion (Ilievski, 2007; OECD Report, 2004). Within this context, the introduction of EMI—first through multilingual institutions such as SEEU—represented an innovative institutional model that aligned with both domestic reconciliation efforts and emerging global norms (Koksal, 2014; OECD Report, 2004).

However, as the country deepened its engagement with European integration and adopted the Bologna reforms, EMI shifted from a conflict-sensitive linguistic compromise to a symbol of international alignment (Galevski, 2021; Zhivkovikj, 2019). This transition marks a key episode in the social process, in which policy actors actively redefined the meaning of English from a tool for internal cohesion to an instrument of external validation. Institutions embraced EMI to comply with new quality assurance frameworks, meet mobility and employability targets, and attract both domestic and international students (Spasovski & Pecakovska, 2020). Thus, EMI evolved from a reactive accommodation strategy into a forward-looking institutional device embedded within a liberalised and competitive higher education environment (Krampf & Heinlein, 2014).

### Steering at a distance and institutional incentives

The concept of steering at a distance offers a useful lens for understanding how EMI became institutionalised without direct national policy mandates (Yuksel et al., 2025). While the state retreated from hands-on control, it exerted influence through quality assurance systems, accreditation processes, and privatisation practices—all of which implicitly favoured institutions that demonstrated innovation, internationalisation, and labour-market relevance.

Universities interpreted these soft pressures through the logic of survival and prestige. In this social context, institutional leaders made specific strategic ‘moves’ to incorporate EMI, ascribing to it the meaning of modernity and global competitiveness (Agai-Lochi, 2015; Gavriilidou & Mitits, 2023). Importantly, this shift was not simply rhetorical; it was enabled by institutional autonomy and legitimised through broader policy narratives about EU accession, employability, and educational excellence (Galevski, 2021). In this way, EMI emerged as an institutional adaptation to externally constructed expectations. SPT shows that this causal pathway relied entirely on how institutional actors made sense of government signals, effectively translating ‘autonomy’ into a mandate for Englishisation.

### Privatisation, symbolism, and institutional competition

The role of privatisation in accelerating EMI growth is particularly notable. Prior research has documented the broader expansion of private higher education in North Macedonia following governance liberalisation (Spasovski & Pecakovska, 2020), but our SPT analysis extends this by identifying the specific mechanism through which privatisation translated into EMI adoption. Our interview and documentary data reveal that newly established private universities actively leveraged EMI as a market differentiator, framing English as both a high-value linguistic resource and a signal of academic prestige to distinguish themselves in an increasingly competitive institutional landscape. This pattern is corroborated by recent institutional analyses of the North Macedonian higher education sector (Sokoli et al., 2025; Bigagli, 2021; Tomovska Misoska et al., 2024).In SPT terminology, private universities utilised EMI as a symbolic ‘move’ to alter the social context, distinguishing themselves from the perceived stagnation of the public sector. This mirrors global trends in EMI adoption as a form of symbolic capital (e.g., Hu, 2009; Jahan & Hamid, 2019); however, the North Macedonian case adds a unique dimension of post-conflict reconciliation and multilingual legitimacy (Czaplinski, 2008).

At the same time, our data show that public universities, despite their longer histories and more bureaucratic governance structures, began to integrate EMI selectively, particularly in programmes aligned with international cooperation or the global job market. This finding diverges from what existing accounts of public sector conservatism in higher education governance might predict (Koksal, 2014; Agai-Lochi, 2015), and suggests that the competitive pressures generated by private sector EMI adoption created a secondary incentive for public institutions to follow, albeit more cautiously.This bifurcation reflects how institutional type, history, and access to external resources shape the uneven development of EMI within a decentralised system (Stojanovski et al., 2018).

### Actor agency and structural constraints

While macro-level reforms provided the ena0bling conditions, the agency of institutional leaders was central to EMI’s implementation. Rectors, vice-rectors, and senior administrators acted as key intermediaries between state objectives, donor agendas, and institutional goals. Their strategic framing of EMI—as a tool for reform, legitimacy, and modernisation—played a critical role in translating abstract policy pressures into concrete academic offerings (Pollozhani, 2022).

However, this agency was not unbounded. Several interviewees acknowledged challenges such as staff preparedness, student language proficiency, and limited infrastructure, particularly in public institutions with fewer resources (Sokoli et al., 2025). These tensions raise important questions about the long-term sustainability and equity of EMI in a linguistically and economically diverse society (Krampf & Heinlein, 2014; Ferguson et al., 2018). This transition raises an important question about the longer-term relationship between EMI and the ethnic harmonisation goals that initially motivated its introduction. In its early instantiation at SEEU, EMI was explicitly embedded within a multilingual, multi-ethnic institutional model designed to foster cross-community interaction and shared academic spaces (Koksal, 2014; OECD Report, 2004). As EMI was progressively decoupled from this reconciliation rationale and reframed as a market differentiator, its potential contribution to inter-ethnic dialogue and social cohesion became secondary to institutional positioning and competitive signalling. Our data do not allow us to assess whether this shift has materially weakened the peacebuilding function of multilingual higher education in North Macedonia, as this fell outside the scope of our interview protocol. However, the trajectory identified in our findings suggests a tension worth monitoring: when the governance logic driving EMI shifts from inclusion to marketisation, the language of instruction risks becoming a mechanism of stratification rather than a bridge between communities, potentially undermining the very social cohesion objectives that first brought English into North Macedonian higher education.

### Implications for language policy and higher education reform

The North Macedonian case offers three core insights. First, our SPT analysis demonstrates that EMI should be understood not as an isolated instructional development but as an emergent governance outcome shaped by the cumulative interaction of intersecting policy domains. In North Macedonia, these domains, i.e., ethnic reconciliation, Europeanisation, and higher education marketization, did not independently produce EMI but created a generative causal sequence in which the institutional conditions established by each episode became the necessary preconditions for EMI expansion in the next. This finding, grounded in our episodic analysis rather than in prior descriptive accounts of the sector, reframes EMI institutionalisation as a by-product of governance transformation rather than a product of linguistic intent (Galevski, 2021).

Second, and most centrally, our analysis identifies the unintended promotion of EMI as the primary mechanism through which governance reforms produced language change in North Macedonia. Our data show that none of the five episodes we traced was designed with EMI as an objective: the OFA aimed at ethnic reconciliation, Bologna harmonisation aimed at European alignment, and privatisation aimed at market liberalisation. Yet each reform, by reconfiguring the governance environment through steering-at-a-distance mechanisms, created structural incentives that institutions interpreted as mandates for Englishisation. This policy spillover dynamic represents the study’s most significant contribution to language policy and planning research, suggesting that the most consequential language policies may be those that contain no explicit reference to language at all.

Third, the North Macedonian case demonstrates that EMI’s relationship to social inequality is neither fixed nor uniform, but is contingent on the governance logic driving its adoption at any given phase. In its early instantiation within the post-conflict multilingual framework of Episode 2, EMI functioned as an inclusionary instrument: embedded within SEEU’s trilingual model, it provided Albanian-speaking and other minority communities with access to internationally recognised higher education that had previously been denied to them, thereby serving goals of ethnic reconciliation and educational equity (Koksal, 2014; OECD Report, 2004). However, as EMI was progressively decoupled from this peacebuilding rationale and reframed as a market differentiator in Episodes 3 and 4, its social effects shifted accordingly. When EMI expansion is driven primarily by privatisation and institutional prestige-seeking rather than by equity goals, access to English-medium programmes becomes stratified by socioeconomic background, concentrating its benefits among students with the financial resources to attend private institutions and the prior linguistic capital to succeed in English-medium environments (Spasovski & Pecakovska, 2020; Stojanovski et al., 2018). This dynamic is consistent with broader patterns of uneven development in which structural advantages tend to reproduce themselves (Ray, 2010). The policy implication is therefore not that EMI is inherently inclusionary or inherently stratifying, but that its social effects are shaped by the governance conditions and institutional logics under which it is adopted—a finding that underscores the importance of contextually sensitive language planning in post-conflict, multilingual societies.

## Conclusion, limitations and future directions

This study has employed SPT to reconstruct the evolution of EMI in North Macedonia as a complex governance outcome rather than a top-down language planning initiative. It identified how macro-level reforms—ranging from the Ohrid Framework Agreement to the implementation of the Bologna Process and the marketisation and privatisation of higher education—created the structural and symbolic conditions for EMI to emerge and expand. By tracing the moves and meanings of key actors, we demonstrated how non-linguistic reforms were interpreted as implicit mandates for Englishisation. Our findings show that EMI was neither planned through a national policy framework nor uniformly adopted across institutions. Instead, it arose from a convergence of external pressures (EU integration, international aid, global rankings) and internal motivations (institutional renewal, market differentiation, reputational signalling). Private universities, in particular, played a pioneering role in embedding EMI, while public universities followed more cautiously, often in response to targeted internationalisation or job market initiatives. The scope and boundaries of these findings are addressed in the limitations and future directions that follow.

While this study provides important insights into the rise of EMI in North Macedonia, its scope is intentionally limited to governance and policy mechanisms. First, the research concentrated on legal documents and interviews with a purposive sample of elite decision-makers (rectors, policymakers, and administrators). We deliberately excluded the perspectives of students and teaching staff, not as an oversight, but because our objective was to trace the macro- and meso-level decision-making processes underlying the adoption of EMI, rather than how it is implemented in the classroom. Future research could fruitfully explore these bottom-up lived experiences to complement our findings on governance.

A further epistemological consideration concerns the reliance on policy documents and elite interviews as primary data sources. As with all retrospective qualitative research, there is an inherent risk that participants’ accounts reconstruct causal sequences in ways that are shaped by knowledge of outcomes, what is sometimes termed ex post rationalisation (Beach & Pedersen, 2019). Similarly, the study does not systematically explore counterfactual scenarios or cases where potential spillover mechanisms did not materialise. We acknowledge these as limitations of the study design. However, two features of our approach mitigate this concern. First, our data triangulation strategy, which included cross-referencing interview accounts with contemporaneous legal documents, policy instruments, and institutional strategies produced at the time of the reforms in question, allows us to assess the consistency between retrospective accounts and the documentation record.

Second, and more fundamentally, our SPT framework, following Kaas et al. (2025), treats the meanings that actors ascribe to reforms as constitutive of the causal process itself rather than as post-hoc distortions of an independently existing causal reality. Within this interpretivist epistemology, the question is not whether actors’ accounts perfectly reflect objective causal mechanisms, but whether those accounts, read alongside documentary evidence, reveal coherent patterns of interpretation and institutional response. Future research employing comparative designs across multiple cases, or incorporating classroom-level and student perspectives, would be well positioned to test the generalisability of the mechanisms identified here and to examine instances where governance reforms did not produce EMI spillover.

Third, regarding generalisability, this single-case study is grounded in North Macedonia’s unique combination of post-conflict governance, multilingual dynamics, and EU accession aspirations. While these specific variables are exceptional, the identified mechanisms, specifically the policy spillover resulting from steering at a distance, possess analytical generalisability (Kaas et al., 2025). The patterns of how non-linguistic reforms drive language shifts may resonate strongly with other multilingual or post-socialist higher education systems, particularly in the Western Balkans.

In sum, the North Macedonian case reveals how EMI can be instrumentalised by institutions navigating a complex policy landscape shaped by global norms, post-conflict reconstruction, and decentralised governance. By demonstrating that EMI often functions as a compliance mechanism and prestige signal rather than a pedagogical strategy, this study highlights the importance of analysing the unintended consequences of governance reforms. Understanding this interplay remains crucial for developing more equitable and context-sensitive approaches to language and education policy.

## Appendix

Semi-Structured Interview Questions (Adapted for North Macedonia)

1. Could you please briefly introduce yourself and your institutional affiliation?
2. Can you describe your academic and/or administrative roles within higher education institutions in North Macedonia?
3. From your perspective as a higher education administrator or academic leader, what do you think are the main drivers behind the introduction and expansion of English-medium instruction (EMI) programmes in North Macedonia?
4. During your time in administrative or academic roles, did you observe any programmes originally taught in Macedonian or Albanian switching to EMI? If so, what factors do you think contributed to this shift?
5. Were there any newly established departments or faculties that were launched directly as EMI programmes? What were the motivations or institutional goals behind these decisions?
6. North Macedonia has undergone a number of governance reforms that promote institutional autonomy alongside accountability mechanisms (e.g., quality assurance agencies, strategic plans). In your view, how has this governance model influenced the adoption of EMI programmes?
7. To what extent did EMI feature in strategic decision-making during your time on governing boards, rectorates, or faculty councils? Were there formal discussions or policy guidelines around this issue?
8. During your term as [insert role], how was the use of English—for teaching, research, or publishing—perceived within your institution? Was there consensus or resistance among academic and administrative staff?
9. Were you aware of any institutional or pedagogical challenges regarding EMI implementation, such as faculty preparedness, student language proficiency, or access to resources? What measures, if any, were taken to support EMI delivery?
10. In your view, does EMI offer an effective and sustainable model for higher education in North Macedonia, particularly given the multilingual and post-conflict context? How do you think it aligns—or clashes—with the role of universities as civic and intellectual institutions?
11. Do factors such as international student recruitment, participation in Erasmus + and other exchange programmes, or collaboration with foreign universities contribute to institutional interest in EMI?
12. Since the early 2000s, North Macedonia has undergone substantial higher education reforms (e.g., Bologna Process, decentralisation, introduction of minority languages in higher education). From your perspective, which of these reforms had the most significant impact on the development of EMI?
13. Is there anything else you would like to add about EMI or language policy in North Macedonian higher education? Are there other scholars or policy actors you believe we should speak with on these issues?
